# Risk Factors for High-Titer Inhibitor Development in Children with Hemophilia A: Results of a Cohort Study

**DOI:** 10.1155/2013/901975

**Published:** 2013-10-02

**Authors:** Susan Halimeh, Christoph Bidlingmaier, Christine Heller, Sven Gutsche, Susanne Holzhauer, Gili Kenet, Karin Kurnik, Daniela Manner, Alfonso Iorio, Ulrike Nowak-Göttl

**Affiliations:** ^1^Gerinnungszentrum Rhein-Ruhr (GZRR), 47051 Duisburg, Germany; ^2^Department of Pediatrics, University Hospital of Munich, 80337 Munich, Germany; ^3^Department of Pediatric Hematology/Oncology, University Hospital of Frankfurt, 60590 Frankfurt, Germany; ^4^Outpatient Hemophilia Treatment Center, 23564 Lubbock, Germany; ^5^Department of Pediatric Hematology/Oncology, Charite, 13353 Berlin, Germany; ^6^The Israel National Hemophilia Centre, Sheba Medical Centre, Tel-Hashomer and the Sackler Medical School, Tel Aviv, 52621 Tel-Hashomer, Israel; ^7^Department of Pediatrics, University Hospital of Münster, 48149 Münster, Germany; ^8^Department of Clinical Epidemiology and Biostatistics, McMaster University, Hamilton, ON, Canada L85 4K1; ^9^Thrombosis & Hemophilia Treatment Center, Institute of Clinical Chemistry, University Hospital of Kiel, 24105 Kiel, Germany

## Abstract

Among the discussed risk factors for high-titre inhibitor (HRI) development in patients with hemophilia A (HA) are high dose FVIII replacement therapy and use of recombinant FVIII concentrates (rFVIII). The aim of this study was to evaluate the aforementioned risk factors for HRI development in children with hemophilia A ≤2%. About 288 ascertained PUPs (Israel and Germany) were followed after initial HA diagnosis over 200 exposure days. Inhibitor-free survival, hazard ratios (HR), and 95% confidence intervals (CIs) were calculated. Adjustment was performed for factor VIII concentrates, median single dose over the first three months of treatment, first FVIII administration before the age of three months, presence of risk HA gene mutations, “intensive treatment moments” and “year of birth” (proxy for different treatment periods). HRI occurred in 71/288 children (24.7%). In multivariate analysis adjusted for “year of birth”, underlying risk gene mutations (HR/CI: 2.37/1.40–3.99), FVIII dose, measured per one IU increase per kgbw (HR/CI: 1.05/1.04–1.07), and first FVIII administration before the age of three months showed a significant impact on HR development. The risk of HRI development was similar for recombinant or plasmatic FVIII products. Children at risk should be treated with carefully calculated lower dose regimens, adapted to individual bleeding situations.

## 1. Introduction

The development of problematic inhibitor antibodies against factor VIII is the most important clinical challenge for patients with hemophilia A (HA) and their treating physicians [1]. Apart from endogenous risk factors for inhibitor development, such as the underlying severity and the underlying hemophilia A—causing gene mutation, particular interests have been focused on the role of risk factors, which can be influenced by the treating institutions [2–5]. Among treatment-related modifiable risks, the use of recombinant FVIII (rFVIII) concentrates or high dose FVIII administration was controversially discussed as potential risk factors for inhibitor development [2–6], and very recently data of the RODIN study pointed out the unexpected finding that the second generation rFVIII products compared to the third generation FVIII concentrates are more immunogenic [7]. However, the paucity of the results of prospective randomized and adequately powered studies still suggest that other individual patient level databases have to be analyzed to overcome some of the controversies discussed.

The primary aim of the present international multicenter study was to evaluate the aforementioned risk factors in an independent cohort of children with severe and moderate-severe hemophilia A, using baseline clinical and laboratory variables. In addition, on an explorative basis the secondary aim of this cohort study was to proof the RODIN hypothesis if the use of second generation rFVIII products also has a higher immunogenicity in our cohort.

## 2. Methods

### 2.1. Ethics

The present database study in consecutively recruited pediatric patients with HA was performed in accordance with the ethical standards laid down in a relevant version of the 1964 Declaration of Helsinki and was approved by the Medical Ethics Committee of the University of Münster, Germany, and the institutional review boards of each study center.

The present study was reported in accordance to STROBE guidelines for observational studies [8].

### 2.2. Inclusion/Exclusion Criteria

**Figure 1:**
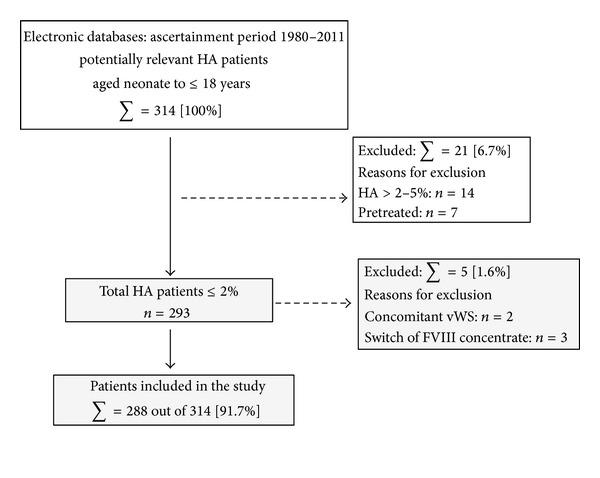
Patient flow chart—inclusion and exclusion criteria are shown.

Inclusion and exclusion criteria are shown in Figure 1. Previously untreated patients (PUPs) with severe and moderately severe hemophilia A (SHA: factor VIII activity ≤2% levels confirmed in at least two independent plasma samples, or via the presence of a high risk gene mutation [4]) aged neonate to ≤18 years at the time of the first presentation in the study center, who had been admitted to the University Children's Hospitals of Frankfurt, Halle, the MVZ Duisburg, Kiel-Lubbock, Munich, Münster, Germany, and the Hemophilia Treatment Center Tel-Hashomer, Israel, at the first symptomatic onset of the disease [9–12]. Patients born before 1980, pediatric patients with HA additionally carrying von Willebrand disease, and children with HA >2% were not included in this cohort study. In addition, children pretreated with transfusion of red blood cell concentrate, fresh frozen plasma before the first administration of factor VIII concentrate, and patients in whom a switch of factor concentrates during the study period was performed were not enrolled. To avoid family cluster effects in both countries, only the first HA patient who presented for diagnosis at the treatment center was included in the present study. 

The final study cohort included 288 consecutively ascertained PUPs with HA ≤2% who were followed over the first 200 exposure days. Prior to enrollment into the German database in each of the study centers, patients were prospectively followed with respect to high-titer inhibitor development. Prior to starting of prophylaxis, patients were followed monthly, and on prophylaxis clinical and laboratory exploration was performed every three to four days until exposure days (ED) 20, at least weekly until ED 50, followed by monthly intervals thereafter in the majority of patients. Twenty-three Israeli patients (8.1%) born between 2001 and 2009 were enrolled in parallel in the RODIN cohort [7] and thus were excluded from the FVIII immunogenicity study (secondary study aim). No further patient overlap has to be reported.

### 2.3. Outcome Measures

#### 2.3.1. Primary Study Aim

High-titer inhibitor-free survival (IFS: first 200 ED) with respect to the discussed possible risk factors.

#### 2.3.2. Secondary Study Aim

High-titer IFS with respect to second generation rFVIII products compared to nonsecond generation rFVIII products (first rFVIII) and pdFVIII.

### 2.4. Study Population

From 1980 to 2011, 314 consecutive pediatric PUPs of Caucasian origin with a first symptomatic onset of HA were ascertained (Figure 1). 

### 2.5. Treatment

At the discretion of the participating centers and according to standard of care in the years of patient enrollment, children were either treated with primary prophylaxis or with on-demand therapy followed by secondary prophylaxis. Treatment in HA patients ≤2% was started in the year of birth. For patients presenting with severe soft tissue bleeding at HA onset, an intensified treatment protocol was introduced in the mid-1990s. These children received a primary prophylactic treatment regimen following the first symptomatic hemorrhage. In cases of trauma-associated or large spontaneous hemorrhage, two-to-three daily FVIII infusions were administered for a minimum of five to seven days. The latter treatment episodes were classified as “intensified treatment moments.”

### 2.6. Factor VIII Products

Apart from pdFVIII products with and without von Willebrand factor content (Beriate P, Factor VIII SDH Intersero, Fanhdi, Haemoctin SDH, Hemophil M, Humate P, Octanate), first generation rFVIII products (Helixate/Kogenate: full-length FVIII, derived from baby hamster kidney (BHK) cell lines, human albumin-stabilized; Recombinate: full-length FVIII, derived from Chinese hamster ovary (CHO) cell lines, human albumin-stabilized) and second generation rFVIII products (Helixate NexGen/Kogenate FS: full-length FVIII, derived from BHK cells, sucrose-stabilized; Refacto: B-domain-deleted FVIII, derived from CHO cells, sucrose-stabilized) were administered due to the discretion of the participating study centers (classification of rFVIII products according to Josephson & Abshire 2004:13). In this study cohort, the plasma-albumin-free third generation rFVIII Advate (full-length FVIII, derived from CHO cells, trehalose-stabilized) was not included into the statistical analysis because only two patients received this product.

### 2.7. Data Collection

Data include baseline factor VIII, factor VIII genotype, age at first factor VIII infusion, factor VIII brand, median single dose administered over the first three months of treatment, frequency of weekly factor administration, type of bleeding requiring intensive FVIII administration (intensive treatment moments, such as intracerebral hemorrhage or surgery) ethnicity, family history of inhibitor development, year of birth, country of patient origin, results of inhibitor measurements, and FVIII ED. 

### 2.8. Laboratory Analysis

Plasma levels of factor VIII were determined by one-stage clotting assays using standard laboratory methods. Inhibitor testing was performed at least monthly when on therapy using the Bethesda method or its modification (Nijmegen). The lower detection limit was set according to the inhibitor assay used in each study center, and a peak inhibitor titer of >5 BU was defined as high responder (HR). A positive HR inhibitor testing was stated when an HR inhibitor was measured at least in two independent follow-up visits. 

Prior to starting of prophylaxis, laboratory assessment of inhibitor status was performed monthly, and on prophylaxis laboratory assessment of inhibitor status was performed every 4-5 ED until ED 20 and thereafter at least weekly until ED 50, followed by monthly intervals thereafter in the majority of patients. Protocols for inhibitor testing were center specific and did not differ among patients treated with various products. 

### 2.9. Statistics

Statistical analyses were performed with the MedCalc software (version 12.3.0) and the StatView 5 software package (SAS Institute Inc.). Continuous variables were presented as median (minimum–maximum) values and evaluated by nonparametric statistics using the Wilcoxon-Mann-Whitney *U* test. Frequency distributions of adverse outcome were compared with chi-square test or, if necessary, Fisher's exact test. In univariate analysis (logistic regression), risk ratios were calculated as odds ratios (ORs)/95% CIs. IFS, defined as the number of cumulative ED until high-titer inhibitor development, was calculated with Cox proportional-hazards regression, and hazard ratios (HRs)/95% confidence intervals (CIs) were calculated additionally. Based on data obtained from (i) a literature research [2–5, 7] and (ii) results from univariate analysis, the following variables were incorporated in the multivariate analysis: first-line use of plasma-derived (pd) or first generation rFVIII products versus second generation rFVIII concentrates, FVIII median single dose per IU/kg bodyweight (bw) applied over the first three months of treatment (proxy for treatment intensity), presence of risk gene mutations ((a) and (b): (a) large deletion and nonsense ⋙ (b) intron 22 inversion) versus mutations with a lower risk ((c) and (d): ≫ (c) missense > (d) small deletions: 4), “presence versus absence” of intensified treatment moments, and “early” (birth to three months of age) versus “late” (>three months) administration of first FVIII concentrate (variables were entered into the model if *P* < 0.1 and removed from the model if *P* ≥ 0.1). In addition, since treatment regimens were modified over time, the model was adjusted for different treatment periods using “year of birth” as proxy. Adjusted risks were expressed as hazard ratios (HRs) and 95% CIs. Using a rule of thumb for proportional hazards analysis of including one independent predictor for approximately 10 outcomes, we were able to include six predictors in the model (71 HR events : 14). The criterion for statistical significance was set at alpha = 0.05. *P* values are based on two-sided test.

## 3. Results 

### 3.1. Study Population

Patient' characteristics are shown in Table 1. According to inclusion and exclusion criteria, the final study cohort ascertained from 1980 to 2011 included 288 PUPs with HA ≤2% available for analysis. Children with FVIII activities <1% did not differ significantly from patients with HA severity between 1% and 2% with respect to clinical phenotypes or genotypes. The median (min–max) single FVIII administered over the first three months of treatment, the median weekly substitution intervals on prophylaxis prior to inhibitor development, and reasons for intensified FVIII treatment are shown in Table 1. The distribution of factor concentrates with respect to pdFVIII, first, second, and third generation rFVIII products is shown in Table 1. 

### 3.2. HRI Development

**Figure 2:**
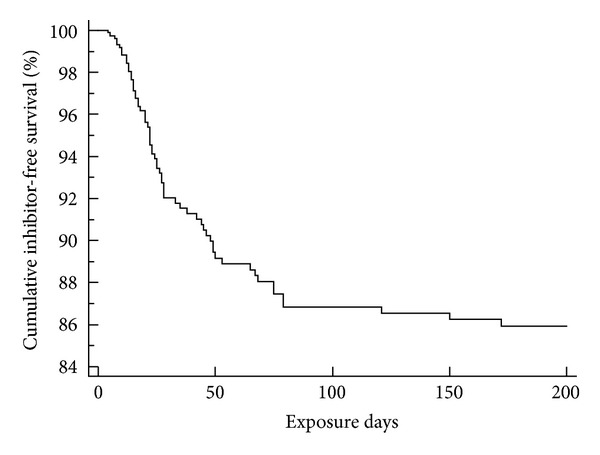
Cumulative inhibitor-free survival in children with severe and moderate severe hemophilia A.

 During the follow-up period (200 ED) in the HA patients investigated, the overall HRI status was 71/288 (24.7%). HRI was diagnosed in 40 of 146 cases (27.4%) with the intron 22 inversion, in 3 of 10 cases (30.0%) carrying large deletions, and in 5 of 16 patients with nonsense mutations (31.3%). Furthermore, persistent HRI was found in 8 of 39 children (20.5%) with missense mutations, in 5 of 34 individuals (14.7%) with small deletions, and in 10 of 43 children (23.3%) in which we did not find one of the above listed variants so far. The corresponding IFS is shown in Figure 2. In patients using second generation FVIII products, the HR-inhibitor rate during the observation period was significantly higher compared to (i) first generation products (50.7% versus 19.5%; *P* = 0.002) and (ii) pdFVIII concentrates (50.7% versus 16.4% *P* < 0.001). Detailed information with respect to the individual factor concentrates used is shown in Table 1. Furthermore, we could not detect statistically significant differences for HRI rates when comparing (i) the use of CHO (full-length; human albumin stabilized) with BHK (full-length; human albumin stabilized) products (*P* = 1.0), (ii) B-domain deleted and full-length (sucrose-stabilized) FVIII products (*P* = 0.32), and (iii) first generation products compared with pdFVIII concentrates (*P* = 0.77). In Table 2, the statistically significant associations between HRI development and the possible predefined risk factors calculated in univariate analysis are summarized. 

### 3.3. Multivariate Analysis (Table 2)

Cox proportional hazards modeling, with the combined variables first-line use of pd or first generation rFVIII products versus second generation rFVIII concentrates (pooling was performed based on results of univariate analysis), FVIII median single dose per IU/kg bodyweight (bw) applied over the first three months of treatment, presence of a risk gene mutation [4], presence versus absence of “intensified treatment moments,” and “early” versus “late” first FVIII administration adjusted for different treatment periods (year of birth) revealed that the development of high titer inhibitors is of multifactorial origin. Apart from the presence of risk gene mutations, the use of high FVIII doses and the administration of “early” FVIII factor concentrate before the age of three months act as modifiers/confounders. Of note, subgroup analysis performed in children with severe HA (*n* = 251) and in children derived from Germany (*n* = 200) did not alter the results obtained from the pooled study group including patients with severe and moderate-severe HA (Supplementary Table 1) (See Supplementary Material available online at http://dx.doi.org/10.1155/2013/901975). In this multivariate analysis, we could no longer find significant risk associations between inhibitor development and the different FVIII products administered or the presence of intensified treatment moments. In addition, when comparing pooled rFVIII products with pdFVIII concentrates, the adjusted HR (95% CIs) was 1.1 (0.6–1.96) and 1.21 (0.58–2.51) when comparing pooled second rFVIII with pdFVIII concentrates. 

## 4. Discussion

The data reported in the present study underline the understanding that HRI development in children with HA ≤2% is of multifactorial origin [2–7, 13–15]. The cumulative HRI rate of 24.7% is within the range expected for database studies [16]. As shown in the subgroup analysis, data in children with severe HA and data obtained from German HA patients only did not substantially alter the results. As secondary study aim we evaluated the recently discussed unexpected observation that second generation rFVIII concentrations are more immunogenic [7]. We investigated the risk of inhibitor development with respect to the use of first versus second generation rFVIII concentrates and subsequently the role of second rFVIII versus pdFVIII products. After exclusion of 23 HA patients from Israel who have been enrolled in parallel into the RODIN study, the higher immunogenicity of second versus first generation rFVIII products and second generation versus pdFVIII concentrates remains statistically significant. We could not detect statistically significant differences between B-domain-deleted and full-length (sucrose-stabilized) FVIII products, nor could we find differences in immunogenicity when comparing the individual first generation FVIII with pdFVIII concentrates. Thus, in the final multivariate analysis we grouped B-domain-deleted with second generation FVIII products and compared this entity with first generation and pdFVIII products. In this multivariate analysis, further controlling for potentially risk factors like “intensive treatment moments” and “early” versus “late” first FVIII administration adjusted for different treatment periods (variable “year of birth”), we could demonstrate that, apart from the genetic HA background, given by the underlying gene mutation [4], the FVIII administration given before the age of three months (variable “early” versus “late” first FVIII administration) did play an independent role in the HRI development in children ascertained from Israel and Germany: when we compared the patients treated with doses between 15 IU/kg and 100 IU/kg, we saw that for every 1 IU/Kg added to dosage, the risk for inhibitor occurrence increased. Thus, when substituting a 6-week-old male (bodyweight five kg) carrying an HA risk mutation with a complete vial of 250 IU of factor FVIII concentrate, that is, 50 IU/kg body weight, the risk to develop persistent HRI is twofold higher compared to an individual using a dose of 25 IU/kg bodyweight (50% mL of a 250 mL vial, littering the remaining 50%). The latter results reflect the real life situation in children with HA. The adjustment variable “year of birth” demonstrated the influence of different treatment periods on HRI development: when we compared patients born between 1980 and 2011, we saw that, for every 1 year added to 1980, the risk for inhibitor occurrence increased. As in the RODIN cohort and our recently published meta-analysis, the risk of high-titer inhibitor development was similar for rFVIII and pdFVIII products [6, 7]. Thus, in light of the recent results of the RODIN study, the positive association between HRI development and second generation FVIII products detected in univariate analysis may be discussed as a chance finding rather than a causal effect.

In the present database study, we were unable to investigate the role of third generation rFVIII concentrates since only two patients received the CHO cell-line derived plasma-albumin-free trehalose-stabilized product in this study. However, the interpretation of less immunogenicity of this plasma-albumin-free product in children with severe or moderate-severe HA should be stated with caution and should be restricted to the recently published RODIN cohort [7], which did not include patients born in 2011. 

Having overcome the problems of transmission of viral diseases, the development of inhibitors currently represents the most challenging issue in patient management of hemophilia today [13, 17]. One important question remains wether different treatment regimens may influence inhibitor development [2–5, 7]. While waiting for prospective data derived from individual birth cohorts to become available [16] and being aware of the limitations of retrospective studies, our aim was to evaluate known risk factors for inhibitor development in severe and moderate-severe HA children on an individual patient-based level. Using baseline clinical and laboratory variables, we have addressed the question of inhibitor development in analyzing data from Israel and Germany, which were collected consecutively over the past thirty years from hemophilia treatment centers [9–12, 15], in which in the majority the same leading physician teams were responsible for patient care. In each of the study centers, patients were prospectively followed with respect to HRI development. 

As in the RODIN study, the approach of this multicenter database study has several limitations: in addition to the limitations discussed by Iorio and coworkers [18] and Kessler and Iorio [19] following the recent report of the RODIN study, (i) the multivariate analysis in the present cohort was performed in Caucasian children derived from Israel and Germany enrolled between 1980 and 2011. As in the RODIN study, this includes a heterogeneous patient population with respect to year of birth, study center, and type of FVIII products. In addition, the pooled patient cohort investigated here is a nonconcurrent cohort study, including inherent biases known for retrospective data collection. However, since in the participating study centers all patients were followed up prospectively with respect to the study endpoint “HRI development,” without differences among patients treated with various FVIII products, reporting, recall, or selection bias is unlikely. (ii) Additionally, the risk profile presented here cannot be generalized to other ethnic groups and needs to be investigated in external non-Israeli/non-German cohorts. (iii) The long recruitment period of this study results in a substantial heterogeneity of treatment strategies and differences in factor VIII products. However, since data were adjusted for different study periods including treatment strategies (variable “year of birth”) and since inhibitor rates were found in line with recently published literature [3–5, 7], selection or reporting bias seems unlikely. As a further limitation, results presented here are restricted to (iv) the development of high responding inhibitors and (v) patients with severe and moderate-severe haemophilia A. Since the number of children treated with each factor product is small, (vi) results of the present cohort study have to be interpreted with caution.

In conclusion, in this multicenter international cohort study we evaluated risk factors, modifiers, and confounders which increase the risk for symptomatic high-titer inhibitor development during hemophilia treatment protocols for children. Until more data are available, children with HA at risk should be treated with rFVIII or pdFVIII concentrates with carefully calculated lower dose regimens, adapted to the individual bleeding situation.

## Supplementary Material

Supplementary Table: Subgroup analysis of patients (1) with severe HA and (2) derived from Germany.

## Figures and Tables

**Table 1:** Characteristics of patients enrolled in the database.

Parameter of interest	Total *N* = 288
Years of birth	1980–2011
Ethnicity: caucasian (%)	100
Factor concentrates used (*n*)	
pdFVIII	177
rFVIII	111
Median (min–max) single dose FVIII (IU/kg/bw)	35 (15–100)
Median (min–max) weekly substitution intervals	3 (1–3)
Persistent high-titer inhibitor	71/288 (24.7%)
pdFVIII*	29/177 (16.38%)
rFVIII	41/111 (36.9%)
(i) First generation:	9/46 (19.5%)
CHO; full-length; human albumin	8/38 (12.5%)
BHK; full-length; human albumin	1/8 (21.0%)
(ii) Second generation:	32/63 (50.7%)
CHO; B-domain-deleted	5/14 (35.7%)
BHK; full-length; sucrose	27/49 (55.1%)
(iii) Third generation	1/2
CHO; full-length; trehalose	1/2 (50.0%)
Indications for intensified treatment	
Total: number	28
Neonatal ICH	6
Cephalhematoma	6
Liver rupture	1
Head/spinal trauma	4
Knee or ankle bleed	4
Tongue bleed	4
Appendectomy	1
Meatotomy	1
Nephroblastoma surgery	1

BHK: baby hamster kidney; BU: Bethesda units; CHO: Chinese hamster ovary; ICH: intracranial hemorrhage; min–max: minimum–maximum; kg bw: kilogram bodyweight; pd: plasma derived; r: recombinant.

*Beriate P (11/50: 22.0%); Hemophil M (8/39: 20.5%); Humate P (10/43: 23.0%).

**Table:** (a)

Parameter investigated	Odds ratio (95% CIs)
Comparator pdFVIII*	
First generation rFVIII (all products)	1.24 (0.54–2.84)
CHO; full-length, human albumin	0.9 (0.39–2.19)
BHK; full-length; human albumin	1.07 (0.45–2.53)
Second generation rFVIII (all products)	2.98 (1.71–5.20)
BHK; full-length sucrose	6.26 (3.14–12.4)
CHO; B-domain deleted	2.83 (0.88–9.07)
*Comparator 1 IU/kg bw *	
Median single FVIII dose increase per one IU/kg bw	1.07 (1.05–1.09)
*Comparator “late” FVIII administration versus *	
“early” FVIII administration	3.17 (1.83–5.51)
*Comparator: intensified treatment moments “absent” *	
Intensified treatment moments present	2.86 (1.18–6.96)
*Comparator *: *risk gene mutation “absent” *	
High risk gene mutation present	5.45 (3.04–9.76)
*Comparator “year of birth” 1 year *	
Increase per birth year	1.10 (1.06–1.15)

kg bw: kilogram bodyweight; *exclusion of 23 Israeli children (RODIN overlap).

**Table:** (b)

Parameter investigated	Hazard ratios (95% CIs)
*Comparator pdFVIII/first generation FVIII *	
Second generation rFVIII	1.37 (0.7–2.68)
*Comparator 1 IU/kg bw *	
Median single FVIII dose increase per one IU/kg bw	1.05 (1.04–1.07)
*Comparator “late” FVIII administration versus *	
“early” FVIII administration	1.97 (1.15–3.4)
*Comparator: intensified treatment moments “absent” *	
Intensified treatment moments present	1.19 (0.58–2.45)
*Comparator: risk gene mutation “absent” *	
High risk gene mutation present	2.37 (1.40–3.99)
*Comparator “year of birth” 1 year *	
Increase per birth year	1.08 (1.03–1.13)

kg bw: kilogram bodyweight.

## Abbreviations

BHK: Baby hamster kidney
BU: Bethesda units
CHO: Chinese hamster ovary
ED: Exposure days
HA: Hemophilia A
HRI: High-titer inhibitor
HR/CI: Hazard ratio/confidence interval
IFS: Inhibitor-free survival
OR/CI: Odds ratio/confidence interval
PUP: Previously untreated patient
pdFVIII: Plasma-derived factor VIII product
rFVIII: Recombinant FVIII product.
