# Supplementary material for: Risk Factors for High-Titer Inhibitor Development in Children with Hemophilia A: Results of a Cohort Study
Source: Biomed Res Int. 2013 Oct 2;2013:901975. doi: 10.1155/2013/901975 (PMC3807559; doi:10.1155/2013/901975)
Supplement: Supplementary file 1 — Supplementary Table: Subgroup analysis of patients (1) with severe HA and (2) derived from Germany. [file 901975.f1.pdf]

Suppl. Table 1a: Multivariate analysis [Cox proportional-hazards regression] in children with severe HA [n=251]: Adjusted hazard ratios [HRs] and 95% confidence intervals [CIs] are shown.

**Parameter investigated**	Hazard ratios **[95% CIs]**
**Comparator pdFVIII/1st generation FVIII**	
2nd generation rFVIII	1.40 [0.71-2.76]
**Comparator 1 IU/kgbw**	
Median single FVIII dose increase per one IU/kgbw	1.05 [1.04-1.07]
**Comparator "late" FVIII administration**	
"early" FVIII administration	1.99 [1.14-3.5]
**Comparator: Intensified treatment moments "absent"**	
Intensified treatment moments present	1.17 [0.56-2.42]
**Comparator "risk gene mutation "absent"**	
high risk gene mutation present	2.17 [1.25-3.75]
**Comparator "year of birth" 1 year**	
Increase per birth year	1.03 [1.03-1.13]

Abbreviations: kgbw: kilogram bodyweight

Suppl. Table 1b: Multivariate analysis [Cox proportional-hazards regression] in German HA patients [n=200]: Adjusted hazard ratios [HRs] and 95% confidence intervals [CIs] are shown.

**Parameter investigated**	Hazard ratios **[95% CIs]**
**Comparator pdFVIII/1st generation FVIII**	
2nd generation rFVIII	2.1 [0.9-4.8]
**Comparator 1 IU/kgbw**	
Median single FVIII dose increase per one IU/kgbw	1.05 [1.03-1.09]
**Comparator "late" FVIII administration**	
"early" FVIII administration	2.51 [1.19-5.3]
**Comparator: Intensified treatment moments "absent"**	
Intensified treatment moments present	1.74 [0.22-13.6]
**Comparator "risk gene mutation "absent"**	
high risk gene mutation present	3.62 [1.64-7.95]
**Comparator "year of birth" 1 year**	
Increase per birth year	1.05 [0.99-1.12]

Abbreviations: kgbw: kilogram bodyweight
